# A Single-Step Method for Harvesting Influenza Viral Particles from MDCK Cell Culture Supernatant with High Yield and Effective Impurity Removal

**DOI:** 10.3390/v16050768

**Published:** 2024-05-13

**Authors:** Sixu Liu, Jingqi Li, Qingtian Cheng, Kangyi Duan, Zhan Wang, Shuang Yan, Shuaishuai Tian, Hairui Wang, Shaobin Wu, Xinkui Lei, Yu Yang, Ningning Ma

**Affiliations:** 1 Wuya College of Innovation, Shenyang Pharmaceutical University, Shenyang 110016, China; liusixu2020@163.com (S.L.); lijingqispu@139.com (J.L.); chengqingtian0101@163.com (Q.C.); duankangyi_spu@163.com (K.D.); yanshuang456@foxmail.com (S.Y.); tianshuaishuai0725@163.com (S.T.); wanghairuispu@163.com (H.W.); shaobinwu1997@163.com (S.W.); xinkui_lei@163.com (X.L.); 2 GenScript (Shanghai) Biotech Co., Ltd., Shanghai 200131, China; 3 School of Life Science and Biopharmaceutics, Shenyang Pharmaceutical University, Shenyang 110016, China; wangzhan6607@163.com; 4 Qilu Pharmaceutical Co., Ltd., Jinan 250104, China; 5 Beijing Zhifei Lvzhu Biopharmaceutical Co., Ltd., Beijing 100176, China

**Keywords:** influenza vaccine, Madin–Darby canine kidney (MDCK), ion-exchange chromatography, viral purification

## Abstract

Influenza vaccines, which are recommended by the World Health Organization (WHO), are the most effective preventive measure against influenza virus infection. Madin–Darby canine kidney (MDCK) cell culture is an emerging technology used to produce influenza vaccines. One challenge when purifying influenza vaccines using this cell culture system is to efficiently remove impurities, especially host cell double-stranded DNA (dsDNA) and host cell proteins (HCPs), for safety assurance. In this study, we optimized ion-exchange chromatography methods to harvest influenza viruses from an MDCK cell culture broth, the first step in influenza vaccine purification. Bind/elute was chosen as the mode of operation for simplicity. The anion-exchange Q chromatography method was able to efficiently remove dsDNA and HCPs, but the recovery rate for influenza viruses was low. However, the cation-exchange SP process was able to simultaneously achieve high dsDNA and HCP removal and high influenza virus recovery. For the SP process to work, the clarified cell culture broth needed to be diluted to reduce its ionic strength, and the optimal dilution rate was determined to be 1:2 with purified water. The SP process yielded a virus recovery rate exceeding 90%, as measured using a hemagglutination units (HAUs) assay, with removal efficiencies over 97% for HCPs and over 99% for dsDNA. Furthermore, the general applicability of the SP chromatography method was demonstrated with seven strains of influenza viruses recommended for seasonal influenza vaccine production, including H1N1, H3N2, B (Victoria), and B (Yamagata) strains, indicating that the SP process could be utilized as a platform process. The SP process developed in this study showed four advantages: (1) simple operation, (2) a high recovery rate for influenza viruses, (3) a high removal rate for major impurities, and (4) general applicability.

## 1. Introduction

Influenza is one of the biggest public health challenges in the world [1]. Vaccination is the most effective way to prevent influenza infections and their related mortality [2]. Traditional influenza vaccines are produced in chicken embryos. However, the traditional chicken embryo processes have several disadvantages, which include a long production cycle, complex operations, a high risk of microbial contamination, potential mutations during the adaptation of viral influenza in chicken embryos, and a tedious scale-up process [3]. Cell culture processes offer faster production, employ simpler cultivation techniques, and carry lower risks of contamination and viral mutation. Furthermore, cell culture processes can be scaled up more effectively to meet the demands of industrial-scale production. However, a challenge in the cell culture process is the increased reliance on subsequent purification [4,5]. At the end of the cell culture process, the cell culture broth contains large amounts of host cell double-stranded DNA (dsDNA) and host cell proteins (HCPs) that might be oncogenic and might pose a challenge to the safety of the vaccines [6]. In addition, due to the high mutation rate of the influenza virus, the purification process must be adaptable to the different viral strains recommended by the World Health Organization (WHO) each year. Therefore, establishing an efficient and universal virus purification process is crucial for the cell culture-based production process.

The first step in the purification process is to separate and concentrate influenza viral particles from clarified allantoic fluid or cell culture supernatant [7]. Harvest methods for chicken-embryo-derived vaccines include precipitation, gel filtration, centrifugation, and ultrafiltration. Precipitation with ethanol, methanol, polyethylene glycol (PEG), or basic calcium dihydrogen phosphate has been reported [8]. Gel filtration has also been reported [9]. However, neither precipitation nor gel filtration has been applied in large-scale productions. The most common harvest method in the production of egg-derived influenza vaccines is density gradient centrifugation. To this day, most manufacturers still utilize this traditional purification method [10].

The purification of cell-culture-derived influenza vaccines needs to meet the increased demand for impurity removal and the requirements of modern GMP practices [11]. Compared to density gradient centrifugation, chromatography-based purification methods offer the benefits of more effective impurity removal and fewer manual operations [12]. Ion-exchange chromatography [13,14], hydrophobic interaction chromatography [12], size-exclusion chromatography [15], and affinity chromatography [6] have been investigated for this purpose. Hydrophobic interaction chromatography and affinity chromatography methods need the cell culture broth to be concentrated, mostly through ultrafiltration, prior to loading. When *Euonymus europaeus* lectin was used as an affinity ligand, the virus recovery in the product fraction was 97% and the depletion rates of dsDNA and total protein were 99.9% and 79%, respectively [16]. Weigel et al. [12] reported a polypropylene glycol group (PPG-600) hydrophobic interaction chromatography method where 91% of viruses were recovered, while 99% of dsDNA and 54% of total protein were removed. A Sepharose Q XL method was used to purify influenza viruses in a flow-through mode, where the virus recovery was 82% and the total protein and dsDNA were reduced to 68% and 1.6%, respectively [17]. Size-exclusion chromatography methods were reported, but their ability to remove impurities was low [17,18]. Besides the aforementioned packed-bed-based chromatograph, membrane adsorbers were investigated to capture viruses. A sulfated cellulose membrane adsorber was able to obtain a yield of 80% in the eluent, where over 71% and 97.5% of the total protein and dsDNA were removed, respectively [19]. Another membrane chromatography method used a zinc-modified iminodiacetic acid membrane that yielded 64% virus recovery, 74% total protein removal, and 93% dsDNA removal [20]. However, none of the above methods can use clarified cell culture broth directly. A pretreatment process is required for buffer exchange and/or concentrating viruses in the cell culture broth. As the influenza virus has a molecular weight over 500 KD [21,22], ultrafiltration with molecular weight cutoffs (MWCOs) lower than 500 KD with different membrane chemistries is used for this purpose [15,23,24]. The need for this additional ultrafiltration step increases production costs and lowers overall yields.

To date, only a few harvest processes have been reported to capture influenza viruses directly from cell culture supernatant. Anion-exchange chromatography on monoliths achieved 89% virus recovery and over 98% dsDNA removal; however, protein removal was low at 52% [25]. Marichal-Gallardo et al. [26] utilized steric exclusion chromatography to achieve 95% virus recovery, while host cell dsDNA and protein depletion were 99.7% and 92.4%, respectively. Nevertheless, viruses needed to be precipitated from the clarified broth using 8% PGE-6000 prior to loading. The pressure was high since PEG binding leads to virus accretion and an increase in viscosity. Kalbfuss et al. [27] utilized Sartobind^®^ anion-exchange membrane adsorbers to bind viruses. The virus recovery was 72% and the total protein removal was 77%, but host cell dsDNA was eluted along with viruses. Other anion-exchange membrane adsorbers were evaluated as well, but the separation of dsDNA from viruses was still poor [13]. After process optimization to increase dsDNA removal, virus recovery was reduced to only 45.8% [13].

This study aimed to develop a simple but efficient purification step that not only captures viruses from cell culture supernatant without a complex pretreatment but also delivers superior performance in terms of virus recovery and host cell dsDNA and protein removal. The harvest process was also intended to be universal so that no significant development work is required when seasonal strain changeovers occur. After screening an array of purification resins, ion-exchange chromatographies were chosen as the focus of this investigation.

## 2. Materials and Methods

### 2.1. Cell Culture and Virus Propagation

Seven influenza virus strains were used in this study (A/Switzerland/9715293/2013 (H3N2) NIB-88, A/Texas/50/2012 (H3N2), A/Hongkong/4801/2014 (H3N2), A/Singapore/INFIMH-16-0019/2016 (H3N2), A/California/7/2009 (H1N1) X179A, B/Brisbane/60/2008 NYMC BX-35 (Victoria), and B/Massachusetts/2/2012-like virus NYMC BX-51B (Yamagata)). They were kindly provided by the Wuhan Institute of Biological Products, China. These strains have been recommended for producing seasonal influenza vaccines by the WHO in the past few years.

Influenza viruses were produced in a clonal Madin–Darby canine kidney (MDCK) cell line that was developed in-house from the parental MDCK CCL-34 cell line (ATCC, Manassas, VA, USA). The clonal MDCK cell line was adapted for serum-free suspension culture. MDCK cells were routinely maintained in a serum-free MDCK 302 medium (Yskbio, Hangzhou, China) at a seeding density of 0.5 × 10^6^ cells/mL and were subcultured every three days. For vaccine production, MDCK cells were grown in a 1:1 mixture of serum-free MDCK 302 and serum-free MDCK 303 media (Yskbio, Hangzhou, China) in 2 L bioreactors (Eppendorf, Hamburg, Germany). When the cell density reached 4 × 10^6^ cells/mL, the cells were infected with the respective strains at a multiplicity of infection (MOI) of ~100. Then, 15 μM N-tosyl-L-phenylalanyl chloromethyl ketone (TPCK)-treated trypsin (Sigma-Aldrich, St Louis, MO, USA) was supplemented. When the cell viability dropped below 50%, the cell culture broths were harvested and inactivated with 0.125‰ β-propiolactone (Aladdin, Shanghai, China) overnight at 4 °C. The inactivated cell culture supernatant was filtered using a 0.22 μm membrane filter (Jinteng, Tianjin, China) and stored at −20 °C.

### 2.2. Ion-Exchange Chromatography

All ion-exchange chromatography experiments were performed on an AKTA purifier 10 liquid chromatography system (Cytiva Corporation, Marlborough, MA, USA). Protein quantification was provided online by a UV detector at a wavelength of 280 nm. The salt concentration/ionic strength was monitored using a conductivity detector. The A/Switzerland/9715293/2013 (H3N2) NIB-88 strain was used to develop and optimize this method, and the six remaining strains were used to validate its general applicability. The flow rate of the whole process was 1 mL/min.

A 5 mL HiTrap Q XL (abbreviated as Q) column (Cytiva Corporation, Marlborough, MA, USA) was used for Q method development. The Q column was equilibrated with 0.1 M NaCl and 20 mM Tris (pH 6.0–9.0). After loading, the column was washed with an equilibration buffer until the UV absorption returned to baseline. The virus particles captured using the column were eluted with 1.5 M NaCl and 20 mM Tris (pH 6.0–9.0). Columns were regenerated with 0.5 M NaOH in all experiments.

A 5 mL HiTrap SP Sepharose FF (abbreviated as SP) column (Cytiva Corporation, Marlborough, MA, USA) was employed to capture influenza virus particles. The adsorption capacity of the ion-exchange chromatography toward influenza virus particles was investigated by changing the pH and ionic strength of the equilibrium buffer and the virus harvest. The pH of the virus harvest and the equilibration buffer was controlled by adding HCl or NaOH. The ionic strength of the supernatants and the equilibration buffer was adjusted with NaCl or ultrapure water. The column was equilibrated with 5 column volumes (CVs) of 0.03–0.1 M NaCl and 20 mM Tris (pH 7.2). After supernatants were loaded onto the column, the equilibration buffer was used to remove unbound impurities. Elution was performed with 0.1–1.5 M NaCl and 20 mM Tris (pH 7.2). The flow rate of the whole process was 1 mL/min. An XK 16/20 column (Cytiva Corporation, Marlborough, MA, USA) packed with 25 mL of SP resins (Huachun, Hangzhou, China) was used for validation. The same process as in the 5 mL prepacked column was used, but the flow rate was increased to 5 mL/min.

### 2.3. Hemagglutination Assay (HA Assay)

An HA assay was used for the quantification of virus titers with 1% guinea pig erythrocytes (Beijing Boehringer Ingelheim, Beijing, China) in phosphate-buffered saline (PBS). First, 25 µL of PBS was loaded in all wells of a U-bottom 96-well microtiter plate (Taizhou Qiangxin, Taizhou, China). Then, 25 µL of the test sample was added to the first line of wells. After mixing, 25 μL was transferred from the first line to the next line, and so on. Finally, 25 µL from the wells in the last line was discarded. PBS was used as a blank control, and 25 µL of 1% guinea pig erythrocytes was added to each well. The plate was incubated at room temperature for 40 min before reading. All samples were measured in duplicate. The results were given in hemagglutination units/50 µL (HAUs/50 µL).

### 2.4. Total Protein Assay

The total protein in each sample fraction was quantified using a Protein Assay Kit (Beyotime, Shanghai, China) according to the manufacturer’s instructions. Samples were transferred to a 96-well microplate (Corning, NY, USA) with bovine serum albumin (Beyotime, Shanghai, China) as a standard. The absorbance was measured at 562 nm using an Infinite^®^ 2000 (Tecan, Männedorf, Switzerland) multifunctional microplate reader. All samples were measured in triplicate.

### 2.5. Total dsDNA Assay

Host cell dsDNA was measured with a PicoGreen dsDNA Quantitation Reagent (Invitrogen, Eugene, OR, USA) according to the manufacturer’s instructions. Briefly, samples were transferred to a black flat-bottom 96-well microplate (Thermo Fisher Scientific, Waltham, MA, USA) with λ-DNA (Invitrogen, Eugene, OR, USA) as a standard and mixed with fluorescent dyes (Invitrogen, Eugene, OR, USA). The fluorescence intensity was measured using an Infinite^®^ 2000 (Tecan, Männedorf, Switzerland) multifunctional microplate reader (~480 nm excitation and ~520 nm emission). All samples were measured in triplicate.

### 2.6. SDS-PAGE

Sodium dodecyl sulfate–polyacrylamide gel electrophoresis (SDS-PAGE) was carried out under non-reducing conditions using an SDS-PAGE Gel Kit (Solarbio, Beijing, China). Coomassie brilliant blue staining (Solarbio, Beijing, China) was used to visualize the proteins.

## 3. Results

### 3.1. Capturing Influenza Virus Particles on Q Resins

**Figure 1:**
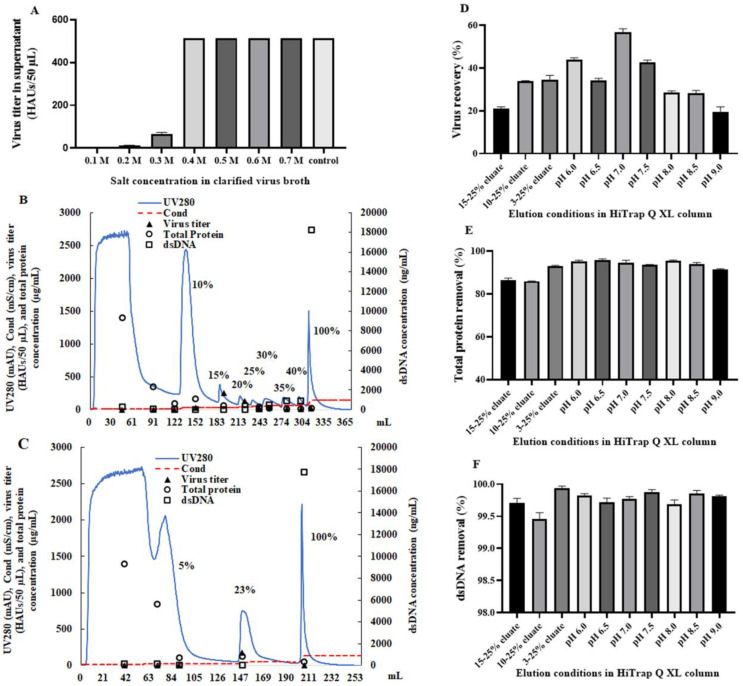
Development of a Q chromatographic method to capture virus particles while removing two major impurities, host cell proteins and dsDNA. (**A**) The static binding of the viruses in the cell culture supernatant to Q resins under different NaCl concentrations. The virus titers in the supernatant were measured using a hemagglutination assay (HA assay). (**B**) Multistep gradient elution at 10% to 100% 1.5 M NaCl. The UV absorbance at 280 nm (—) and the conductivity (– –) were monitored online. The virus titer (▲), dsDNA concentration (□), and total protein concentration (○) in each fraction were measured offline. (**C**) Three-step gradient elution at 5%, 23%, and 100% 1.5 M NaCl. The UV absorbance at 280 nm (—) and the conductivity (– –) were monitored online. The virus titer (▲), dsDNA concentration (□), and total protein concentration (○) in each fraction were measured offline. The virus recovery (**D**), total protein removal (**E**), and dsDNA removal (**F**) in the 23% eluate (**C**) and in the pH optimization study. The error bars in A, D, E, and F represent the standard error of the mean (n = 2). HAUs: hemagglutination units.

We first investigated the binding of the influenza viruses (A/Switzerland/9715293/2013 (H3N2) NIB-88) in the cell culture supernatant on Q resins at different ionic strengths. The ionic strength of the cell culture harvest was close to that of 0.1 M NaCl. Additional NaCl was supplemented into the supernatant to adjust its ionic strength to that of 0.2 M, 0.3 M, 0.4 M, 0.5 M, 0.6 M, and 0.7 M NaCl. The supernatants were statically incubated with Q resins at room temperature overnight. A negative control, where no resins were present in the supernatant, was used for comparison. The virus titers in the supernatant were measured. As shown in Figure 1A, at 0.1–0.3 M NaCl, most viruses were absorbed into Q resins, while at 0.4 M NaCl and beyond, all virus stayed in the supernatant. The static absorption study indicated that the cell culture supernatant could be loaded directly on Q resins for virus particle absorption.

The elution condition was investigated with a Q column, where the equilibration buffer was 0.1 M NaCl and 20 mM Tris (pH 7.2) (buffer A) and the elution buffer was 1.5 M NaCl and 20 mM Tris (pH 7.2) (buffer B). After loading the cell culture supernatant into the Q column, a multistep gradient elution was carried out with 10%, 15%, 20%, 25%, 30%, 35%, 40%, and 100% buffer B (Figure 1B). Host cell proteins were mostly eluted at 10%, while viruses were eluted at 15% and dsDNA was eluted at 100% buffer B. Subsequently, the gradient of buffer B was fine-tuned to reveal the proper conditions for eluting viruses, host cell proteins, and dsDNA. Ultimately, the step-wise elution was simplified to 5%, 23%, and 100% buffer B, and good separation of virus particles from host cell proteins and dsDNA was achieved (Figure 1C). In the 23% eluate, 94% of host cell proteins and 99.8% of dsDNA were removed (Figure 1E,F), but the recovery of viruses was low at 35% (Figure 1D).

The elution pH was optimized in the range of 6.0–9.0 to improve recovery. Across the whole test range, the removal of total protein was above 90% (Figure 1E) and the removal of dsDNA was above 99% (Figure 1F). The highest recovery was achieved at pH 7.0. However, it was still lower than 60% (Figure 1D).

### 3.2. Capturing Influenza Virus Particles on SP Resins

**Figure 2:**
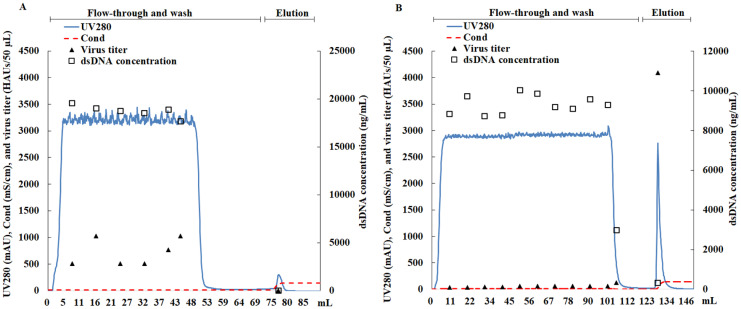
Diluting the supernatant allowed virus particles to bind to the SP resins. (**A**) Viruses flowed through the SP resins with dsDNA when the clarified cell culture broth was directly loaded. Equilibration buffer: 0.1 M NaCl and 20 mM Tris (pH 7.2); loading: 50 mL of clarified broth. (**B**) Viruses bound to the SP resins and separated well from dsDNA after a 1:1 dilution of the cell culture supernatant with ultrapurified water. Equilibration buffer: 0.05 M NaCl and 20 mM Tris (pH 7.2); loading: 100 mL samples (50 mL of clarified broth diluted with 50 mL of ultrapure water). Throughout the whole process, the flow rate was controlled at 1 mL/min and the pH was 7.2. The UV absorbance at 280 nm (—), conductivity (– –), virus titer (▲), and dsDNA concentration (□) are shown. HAUs: hemagglutination units.

The same influenza strain as in the Q study, A/Switzerland/9715293/2013 (H3N2) NIB-88, was used in the development of an SP-based harvest process. As shown in Figure 2A, the viruses in the cell culture supernatant did not bind to SP resins and flowed through an SP column. The equilibration buffer (buffer A) and elution buffer (buffer B) were the same as those used in the Q study. The virus titer in the eluent was only 4 HAUs/50 µL, while the titer in the flow-through (8–44 mL) was between 512 and 1024 HAUs/50 µL (Figure 2A). As a goal of this study was to develop a binding/elution process, the binding of virus particles to SP resins was increased by lowering the ionic strength of the supernatant. By diluting the supernatant with ultrapure water at 1:1, viruses could be captured by SP resins and eluted by buffer B (Figure 2B). It should be noted that to match the diluted load, the concentration of NaCl in buffer A was reduced by half to 0.05 M. The virus titer in the eluent was 4096 HAUs/50 µL (Figure 2B), which was eight-fold higher than that in the load. The virus titer in the flow-through (10–105 mL) was low at 64 HAUs/50 µL or lower (Figure 2B). The DNA mostly stayed in the flow-through, and only a low concentration of DNA was present in the eluent, achieving good separation of DNA and virus particles.

**Figure 3:**
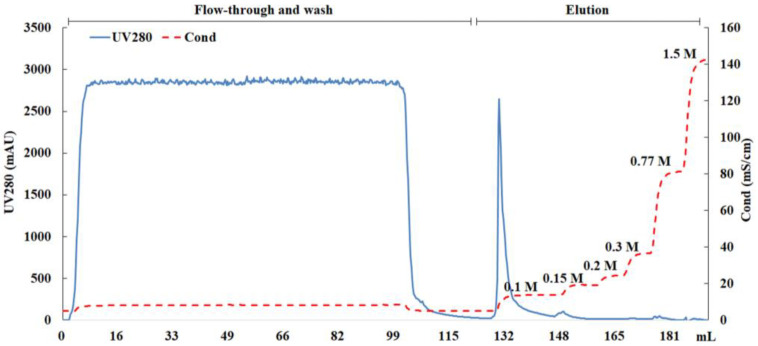
Optimization of SP elution buffer. Equilibration buffer A: 0.05 M NaCl and 20 mM Tris (pH 7.2); elution buffer B: 1.5 M NaCl and 20 mM Tris (pH 7.2). The eluate gradient was gradually increased to 0.1 M/0.15 M/0.2 M/0.3 M/0.77 M/1.5 M NaCl. Throughout the whole process, the flow rate was controlled at 1 mL/min and the pH was 7.2. The UV absorbance at 280 nm (—) and conductivity (– –) are shown.

The elution condition was subsequently optimized. With the same 1:1 dilution of the cell culture supernatant, a step elution was carried out with buffer B, as indicated in Figure 3. Viral particles were eluted with 0.1 M NaCl. Consequently, the NaCl concentration in buffer B was reduced from 1.5 M to 0.1 M in subsequent experiments. The lower salt concentration in the eluent had the additional benefit of eliminating the need for a buffer exchange step to reduce the conductivity for the subsequent purification process.

The dilution ratio was studied in the range of 1:0 to 1:3. In this study, the virus titer, total protein concentration, and dsDNA concentration were measured in the load, flow-through, and elution sections. It should be noted that the total protein was used to approximate host cell proteins, as the viral protein content in the cell culture broth was very low. The recovery of viruses, total protein, and dsDNA was quantified. Again, without dilution, there was almost no virus recovery. At 1:1 dilution, the recovery was 56% (Table 1). The recovery increased to around 100% in the range of 1:1.7 to 1:3, where the viruses in the flow-through were undetected or at very low concentrations. The total protein and dsDNA recovery in the eluents increased at higher dilution ratios, but the levels were low across the whole dilution range (lower than 3% and 1% for total protein and dsDNA, respectively), indicating good separation of viruses from host cell proteins and dsDNA. A 1:2 dilution was chosen as the optimal dilution ratio for the subsequent experiments.

In previous experiments, the loading volume was 10 CVs of clarified cell culture broth prior to dilution. The flow rate was 1 mL/min during the whole purification process. The dynamic binding capacities and separation capabilities of SP resins were studied at different flow rates. Firstly, the flow rate had a significant impact on virus recovery. When the flow rate was increased from 1 mL/min to 5 mL/min, virus recovery decreased from 98.7% to 80.8% (Table 2), while the overall separation of viruses from host cell proteins and dsDNA remained satisfactory. The total protein and dsDNA contents in the eluent decreased slightly, and the rates of removal of the total protein and dsDNA in the eluent were more than 97% and 99%, respectively (Table 2).

When the loading volume tripled from 10 CVs to 30 CVs, there was little difference in the recovery rates of the virus particles, total protein, and dsDNA (Table 2). At 25 and 30 CVs, the recovery rate for viruses remained above 95%, while the rates of removal of the total protein and dsDNA in the eluent remained higher than 97% and 99%, respectively. No loading volume over 30 CVs was investigated, as with a 1:2 dilution 30 CVs corresponded to a nearly 8 h loading time.

### 3.3. Verification of the Optimized SP Process in a Packed 25 mL Column

**Figure 4:**
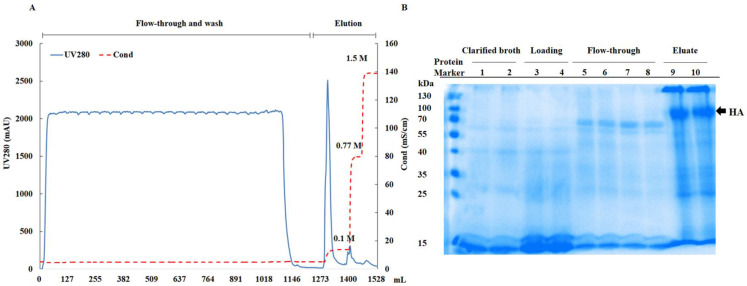
Verification of the SP process with a packed 25 mL SP column. (**A**) Chromatography of the verification study. Equilibration buffer A: 0.03 M NaCl and 20 mM Tris (pH 7.2); elution buffer B: 0.1 M NaCl and 20 mM Tris (pH 7.2); flow rate: 5 mL/min. The UV absorbance at 280 nm (—) and conductivity (– –) are shown. (**B**) Non-reducing SDS-PAGE analysis of chromatographic samples. The clarified cell culture broth was concentrated 5-fold, the loading and flow-through were concentrated 10-fold, and the eluent was untreated prior to loading onto the SDS-PAGE gel. HA: hemagglutinin.

In this study, 25 mL SP resins were packed into an XK 16/20 column and the optimized process described below was verified. Loading: 375 mL (15 CVs) of virus harvest diluted with 750 mL of ultrapure water (pH 7.2). Flow rate: 5 mL/min. Equilibration buffer A: 0.03 M NaCl and 20 mM Tris (pH 7.2). Elution buffer B: 0.1 M NaCl and 20 mM Tris (pH 7.2). The virus particles on the SP resins were eluted with elution buffer B containing 0.1 M NaCl, followed by 0.77 M and 1.5 M NaCl. The performance was very similar to that of the 5 mL prepacked column (Figure 4A). The recovery rate for viruses was 95.3%, and the removal rates for total protein and dsDNA were 97% and 99%, respectively (Table 2). Chromatographic samples were analyzed for protein composition on an SDS-PAGE gel under non-reducing conditions (Figure 4B). The eluent contained predominantly influenza virus proteins, especially hemagglutinin (HA) proteins, while in the flow-through, only proteins unrelated to influenza viruses were observed.

### 3.4. General Applicability of the SP Capture Process to Different Virus Strains

The potential applicability of the SP process as a platform process for different influenza strains was tested. Seven influenza viruses representing all four subtypes or lineages among the quadrivalent seasonal influenza viruses were used, including four H3N2 subtypes (A/Switzerland/9715293/2013 (H3N2) NIB-88, A/Texas/50/2012 (H3N2), A/Hongkong/4801/2014 (H3N2), and A/Singapore/INFIMH-16-0019/2016 (H3N2)), one H1N1 subtype (A/California/7/2009 (H1N1) X179A), and two B lineages (B/Brisbane/60/2008 NYMC BX-35 (Victoria) and B/Massachusetts/2/2012-like virus NYMC BX-51B (Yamagata)).

Table 3 shows that there were no significant differences in the SP cation resin’s ability to capture different influenza virus strains. The virus recovery rates were all above 90%, while the removal rates of total protein and dsDNA were all over 97% and 99%, respectively.

## 4. Discussion

Various chromatographic methods have been studied to purify influenza virus particles, including size-exclusion chromatography, hydrophobic interaction chromatography, affinity chromatography, and ion-exchange chromatography [28].

Affinity chromatography methods like immobilized metal affinity chromatography have strong strain dependency for viral adsorption [20]. Sulfated cellulose affinity chromatography captured H1N1, H3N2, and BV with significantly different rates of HA recovery and impurity removal [29], and its versatility remains to be verified [30]. The a-galactose-specific *Euonymus europaeus* lectin affinity chromatography can capture different influenza virus strains but is only suitable for MDCK-cell-derived viruses [23]. Size-exclusion chromatography and hydrophobic interaction chromatography have limited ability to remove dsDNA and HCPs [12,17,31]. Previous studies have shown that ion-exchange chromatography can be used to harvest influenza viruses, but flow-through mode has frequently been used, which has low HCP-removal efficiency [14,25,27,31,32,33].

In this study, we investigated using both Q and SP resins to harvest influenza viruses. Both the Q and SP methods could remove HCPs and dsDNA efficiently, but the SP method showed much higher recovery of influenza viruses. This can be attributed to the desorption of influenza viruses off Q and SP resins. Influenza viruses were eluted quickly using an SP column under a low salt concentration, while they were eluted gradually from Q resins in a wide range of salt concentrations. The desorption property of anion exchangers for influenza viruses is consistent with the observation of Banjac et al. [32], who attributed this behavior to the variable sizes and compositions of influenza viruses. Another plausible explanation for the higher recovery of the SP method is the weaker binding of influenza viruses on SP resins, where 0.1 M NaCl is sufficient to desorb influenza viruses.

The pH values used to capture the influenza viruses on both the Q and SP resins were in the same range. These results were observed with influenza viruses [32] and β-lactoglobulin A and B (LgA and LgB) [34]. Membrane proteins of influenza viruses have pockets with different amino acid compositions and isoelectric points that may result in different charge distributions on their outer surfaces at the same pH, allowing viral particles to adsorb to both Q and SP. The salt concentration is the major influencing factor in cation-exchange chromatography. To capture the influenza virus, the salt concentration was first investigated. The SP resins need a lower salt concentration than the virus harvest, so we diluted the harvest with ultrapure water at 1:2 so that almost all viruses could be captured. The adsorption of viruses with this method from cell culture supernatant directly improves virus recovery and productivity without ultrafiltration or a dialysis pretreatment. We also surveyed the dynamic binding capacity of SP resins. As the flow rate increased, although the removal of impurities improved slightly, the virus recovery decreased. Considering the purification time, the highest loading volume was 30 CVs, and the recovery rate of the virus remained above 90%. Studies have shown that SP resin has a strong binding capacity and that its viral load is higher than those observed for other ion-exchange resins [32].

The SP method is sensitive to the ionic strength at loading and the flow rate. Viral particles in a cell culture supernatant cannot be captured by SP resins. A simple procedure that involves diluting the supernatant with ultrapure water allows nearly all viral particles to be captured. The optimal dilution rate was determined to be 1:2. This dilution procedure could be achieved in a large-scale production using in-line dilution with water for injection (WFI). Flow rates higher than 1 mL/min through a prepacked 5 mL column, which correspond to a linear flow rate of approximately 0.5 cm/min, result in lower virus recovery. The low flow rate is a major limitation of the SP process, especially considering that the load is diluted three-fold.

The versatility of the influenza virus purification process is of great significance. This process has to be applied to all strains recommended annually by the WHO, with only limited tinkering. In this study, seven influenzas A and B strains were tested, and all seven demonstrated satisfactory performance. This was different from previous publications. Banjac et al. [32] utilized cation-exchange chromatography, CIM SO3, to capture four recombinant influenza virus strains. One strain, ΔNS1-FLUB, showed low binding capability. 

## 5. Conclusions

Two ionic exchange methods were developed to capture influenza virus particles from a cell culture supernatant, one based on anion-exchange resin Q and the other based on cation-exchange resin SP. Both methods were operated in bind/elute mode, and both methods were able to efficiently remove host cell proteins and dsDNA from viral particles. The recovery of the Q method was low at 56% of the maximum, while that of the SP method was generally over 90% or even 95%. Hence, the SP method was chosen for the harvest of viral particles from an MDCK cell culture broth. For the SP method, the rates of removal of host cell proteins and dsDNA were over 97% and 99%, respectively. The bind/elute SP method was only feasible after the cell culture supernatant was diluted with water to reduce its conductivity. Almost all viral particles could be captured on an SP column with a dilution ratio between 1:1.7 and 1:3.

The SP method was confirmed to be universally applicable to other influenza virus strains. Seven strains, including H1N1, H3N2, B (Yamagata), and B (Victoria), were tested, and all showed satisfactory viral particle recovery and impurity removal.

The SP method developed in this study is simple to operate, requires no buffer-exchange step before or after the procedure, and is highly efficient for viral particle recovery and impurity removal. It will reduce the reliance on subsequent polishing steps.

In this study, we used the total protein concentration to approximate the host cell protein concentration, as the viral protein content in the cell culture broth was very low. In the eluent, viral proteins accounted for a much larger portion of the total protein. The removal of host cell proteins could be underestimated, but it would not change the conclusion of this study.

## Figures and Tables

**Table 1:** The recovery of viruses, total protein, and dsDNA in the eluent of SP chromatography using clarified cell culture broths with different dilution ratios. The NaCl concentrations in buffer A were 0.1 M, 0.05 M, 0.04 M, 0.035 M, 0.03 M, and 0.025 M for the 1:0, 1:1, 1:1.5, 1:1.7, 1:2, and 1:3 dilution ratios, respectively. The NaCl concentration in buffer B was 0.1 M. Throughout the whole process, the flow rate was controlled at 1 mL/min and the pH was 7.2.

	Loading				Flow-Through		Elution				Recovery (%)		
Dilution Ratio ^a^	Volume (mL)	Virus Titer (HAUs/50 µL)	Total Protein ^b^ (μg/mL)	dsDNA ^c^ (μg/mL)	Volume (mL)	Virus Titer (HAUs/50 µL)	Volume (mL)	Virus Titer (HAUs/50 µL)	Total Protein ^b^ (μg/mL)	dsDNA ^c^ (ng/mL)	Virus	Total Protein	dsDNA
1:0	50	1024	2554.9	18.5	50	1024	2	4	22.5	19.6	0.02	0.035	0.0042
1:1	100	512	1308.5	9.4	100	64	7	4096	274.3	318.2	56.0	1.46	0.24
1:1.5	125	448	1127.6	7.5	125	7	7	6144	354.6	564.5	76.8	1.76	0.42
1:1.7	135	448	1011.4	6.7	135	2	7.5	6144	379.2	825.4	99.5	2.08	0.68
1:2	150	320	894.3	6.2	150	0	8	6144	403.6	1043.2	102.4	2.41	0.89
1:3	200	256	653.7	4.6	200	0	8	6144	411.4	1059.4	96.0	2.52	0.93

^a^ The dilution ratio of virus harvest and ultrapure water. ^b^ Concentration of total protein. ^c^ Concentration of dsDNA. HAUs: hemagglutination units.

**Table 2:** Test of the dynamic binding capacity of SP resins for the purification of virus particles. Equilibration buffer A: 0.03 M NaCl and 20 mM Tris (pH 7.2). Elution buffer B: 0.1 M NaCl and 20 mM Tris (pH 7.2). Dilution ratio: 1:2.

Flow Rate (mL/min)	Volume of SP Resins (mL)	Loading Volume of Clarified Broth ^a^ (CVs)	Recovery (%)		
			Viruses ^b^	Total Protein ^b^	dsDNA ^b^
1	5	10	98.7	2.39	0.88
2	5	10	93.3	2.29	0.78
3	5	10	85.6	1.89	0.58
5	5	10	80.8	1.45	0.26
1	5	20	98.5	2.58	0.84
1	5	25	96.2	2.46	0.88
1	5	30	99.2	2.59	0.90
5	25	15	95.3	2.48	0.91

^a^ The true loading volume was 3 times the volume of the cell culture broth as a result of 1:2 dilution with ultrapurified water. ^b^ Means of two experiments. CVs: column volumes. SP: HiTrap SP Sepharose FF.

**Table 3:** The general applicability of SP resins for the purification of different virus strains.

Influenza Virus Strains	Recovery (%)		
	Viruses ^b^	Total Protein ^b^	dsDNA ^b^
A/Switzerland/9715293/2013 (H3N2) ^a^	98.7 ^a^	2.39 ^a^	0.88 ^a^
A/Texas/50/2012 (H3N2)	95.7	2.45	0.97
A/Hongkong/4801/2014 (H3N2)	97.4	2.21	0.84
A/Singapore/INFIMH-16-0019/2016 (H3N2)	94.6	2.47	0.79
A/California/7/2009 (H1N1) X179A	99.8	2.38	0.86
B/Brisbane/60/2008 NYMC BX-35 (Victoria)	90.1	2.03	0.89
B/Massachusetts/2/2012-like virus NYMC BX-51B (Yamagata)	96.6	2.59	0.92

^a^ Same data as Table 2, line 1. ^b^ Means of two experiments. Loading volume: 10 column volumes. Flow rate: 1 mL/min. Equilibration buffer A: 0.03 M NaCl and 20 mM Tris (pH 7.2). Elution buffer B: 0.1 M NaCl and 20 mM Tris (pH 7.2).

## Data Availability

The data presented in this study are available on request from the corresponding author.
